# Immune System and DNA Repair Defects in Ovarian Cancer: Implications for Locoregional Approaches

**DOI:** 10.3390/ijms20102569

**Published:** 2019-05-25

**Authors:** Alberto Farolfi, Giorgia Gurioli, Paola Fugazzola, Salvatore Luca Burgio, Claudia Casanova, Giorgia Ravaglia, Amelia Altavilla, Matteo Costantini, Andrea Amadori, Massimo Framarini, Luca Ansaloni, Ugo De Giorgi

**Affiliations:** 1 Department of Medical Oncology, Istituto Scientifico Romagnolo per lo Studio e la Cura dei Tumori (IRST) IRCCS, Meldola 47014, Italy; alberto.farolfi@irst.emr.it (A.F.); salvatore.burgio@irst.emr.it (S.L.B.); amelia.altavilla@irst.emr.it (A.A.); ugo.degiorgi@irst.emr.it (U.D.G.); 2 Biosciences Laboratory, Istituto Scientifico Romagnolo per lo Studio e la Cura dei Tumori (IRST) IRCCS, Meldola 47014, Italy; 3 General and Emergency Surgery, Maurizio Bufalini Hospital, Cesena 47521, Italy; paola.fugazzola@auslromagna.it (P.F.); luca.ansaloni@auslromagna.it (L.A.); 4 Oncology Department, Santa Maria delle Croci Hospital, Ravenna 48121, Italy; claudia.casanova@auslromagna.it; 5 Unit of Biostatistics and Clinical Trials, Istituto Scientifico Romagnolo per lo Studio e la Cura dei Tumori (IRST) IRCCS, Meldola 47014, Italy; giorgia.ravaglia@irst.emr.it; 6 Pathology Unity, Morgagni-Pierantoni Hospital, Forlì 47121, Italy; matteo.costantini@auslromagna.it; 7 Department of Gynecological, Morgagni-Pierantoni Hospital, Forlì 47121, Italy; dott.amadori@gmail.com; 8 Department of General Surgery, Morgagni-Pierantoni Hospital, Forlì 47121, Italy; massimo.framarini@auslromagna.it

**Keywords:** DNA repair defects, inflammation, immune system, immunotherapy, ovarian cancer, PARP-inhibitors

## Abstract

In the last few years, substantial progress has been made in the treatment of ovarian cancer, with increased knowledge about the biology of the disease. Ovarian cancer is a neoplasm strongly linked to defects in DNA repair mechanisms, where deficiency in the homologous recombination (HR) system results in a better response of ovarian cancers to therapy, whether platinum-based chemotherapy, anthracyclines, or poly (ADP-ribose) polymerase (PARP) inhibitors. More recently, it has been demonstrated that different ovarian cancer histotypes may have different immunogenicity. Interestingly, defects in HR systems are associated more frequently with higher tumor infiltrating lymphocytes, providing a rationale for developing combination therapy with immune-modulating agents and PARP inhibitors. Again, locoregional therapies combining heat shock and chemotherapy delivery have been shown to induce an anticancer immune response in vitro. Thus, the potential for locoregional therapeutic approaches that may impact the immune system, perhaps in combination with immune-modulating agents or PARP inhibitors, needs to be further explored. With this premise, we reviewed the main biological and clinical data demonstrating a strict interplay between the immune system, DNA repair mechanisms, and intraperitoneal therapies in ovarian cancer, with a focus on potential future therapeutic implications.

## 1. Introduction

Ovarian cancer is the second cause of death from gynecological malignancies, and the seventh most common cause of cancer death worldwide [1]. The median progression-free survival (PFS) and overall survival (OS) for advanced ovarian cancer range between 12 and 24 months and 29 and 65 months, respectively [2,3]. The most common ovarian neoplasm is a high-grade serous histological subtype, accounting for about 70% of cases and causing the majority (90%) of ovarian cancer deaths. 

Other histologic subtypes include low-grade serous, endometrioid, clear-cell, and mucinous ovarian cancers (Table 1) [4,5,6]. Mucinous ovarian cancer is a rare tumor, probably accounting for 3% of all epithelial ovarian cancers. Stage III or IV mucinous ovarian cancer patients have a poorer prognosis than women with other, more common subtypes (particularly serous or endometrioid ovarian cancer), and may be related to a poorer response to chemotherapy [7].

High-grade serous ovarian cancer is frequently associated with DNA repair deficiencies [8]. Alterations in DNA repair pathways represent a common feature of carcinogenesis, as they can drive malignant transformation with the accumulation of genomic alterations in cancer cells [9]. Conversely, the presence of multiple DNA repair systems allows cancer cells to have a compensating mechanism to avoid non-viable amounts of genotoxic stress that would ultimately lead to cell death [10]. 

In around 18% of ovarian cancer patients, it is possible to identify germline mutations in *BRCA1* and *BRCA2*, especially in those with high-grade serous carcinoma [11,12]. When combined with *BRCA* deficiencies resulting from somatic mutations or epigenetic silencing, it appears that up to half of all high-grade serous ovarian cancers have a *BRCA* dysfunction [13,14,15,16]. About 10%–14% of ovarian endometrioid carcinomas present deficiencies in mismatch repair proteins by immunohistochemistry, accounting for the microsatellite instability phenotype [17]. Conversely to high-grade serous ovarian cancer, mucinous ovarian cancers are not associated with *BRCA* mutations or defects in homologous recombination. The most frequent alterations are *KRAS* mutations (in 40% to 65% of cases), *c-MYC* amplifications (65% of cases), *HER2* amplifications (20% to 38% of cases), and *TP53* mutations (50% to 75% of cases). In addition, other alterations have been identified at lower frequencies, such as homozygous deletions in *CDKN2A/B* (in 25% of cases), mutations in *PI3KCA* (13%), and mutations in *PTEN*, *BRAF*, *FGFR*, *KIT*, or *STK11* (2% to 5% of cases) [18].

Although the tumor stage, residual disease after surgical debulking, response to chemotherapy, and *BRCA1/2*-mutation status all affect the outcome of ovarian cancer, the variability in PFS and OS among patients with similar clinical and pathological characteristics makes it difficult to reliably predict outcome. In 2003, Coukos and coworkers reported for the first time that the presence of tumor-infiltrating lymphocytes (TIL) CD3+ correlated with improved clinical outcome in advanced ovarian carcinoma [19].

Peritoneal carcinomatosis (PC) is the major cause of treatment failure in the management of ovarian cancer. However, the finding that ovarian-related PC is a regional disease rather than a systemic disease has led to the development of locoregional approaches to improve ovarian cancer patient outcome. In this context, intraperitoneal chemotherapy, endowing direct exposure of chemotherapy to the peritoneal surface plus intravenous chemotherapy, compared with intravenous chemotherapy alone after complete or optimal primary cytoreductive surgery (CRS), showed a 16-month increase in survival. Nevertheless, this strategy is limited in clinical practice by increased side effects, including catheter-related complications, and the inconvenience of administering therapy intraperitoneally [3]. Even if intraperitoneal chemotherapy has shown benefits as a primary treatment of ovarian cancer [3], this route of administration has not been explored carefully in the context of interval CRS. The results of the randomized trial of interval CRS, with or without hyperthermic intraperitoneal chemotherapy (HIPEC), in ovarian cancer patients who had at least stable disease after three cycles of neoadjuvant chemotherapy with carboplatin and paclitaxel, demonstrated a median overall survival longer in the surgery-plus-HIPEC group than in the surgery group by nearly a year (45.7 versus 33.9 months; HR = 0.67; 95% CI, 0.48 to 0.94; stratified *p* = 0.02) [20,21]. Interestingly, hyperthermia has been shown to increase the cytotoxic effect of cisplatin in preclinical trials, by increasing the tumor penetration, promoting DNA cross-linking, impairing DNA repair pathways, and consequently promoting apoptosis [22].

Thus, in this review, we analyze the strict interplay between DNA repair pathway alterations, the immune system, and inflammation in an attempt to identify unique challenges and opportunities for new treatment strategies.

## 2. DNA Repair Systems

Potentially harmful agents, comprising oxidative stress, ultraviolet light and ionizing radiation, and the use of alkylating and anti-tumor agents, continuously interact with human DNA. Five DNA repair mechanisms are exploited by cells: base excision repair (BER), mismatch repair (MMR), nucleotide excision repair (NER), homologous recombination (HR), and non-homologous end-joining (NHEJ). We briefly described the main characteristics of DNA repair mechanisms below.

BER protects against single-base DNA damage caused by spontaneous depurinations, methylating and oxidizing agents, or other genotoxicants [23]. BER consists of the removal of damaged bases by DNA glycosylases. There are 11 of these enzymes in humans, and each identifies specific lesions; they bind the altered deoxynucleoside in an extrahelical position and catalyze the cleavage of the base–sugar bond. APE-1 is a protein with an endonuclease activity that makes a 5’ nick in the DNA structure and a 3’ hydroxyl that is recognized by DNA repair polymerase β. Poly (ADP-ribose) polymerase-1 (PARP1) binds to the 5’ nick, acting as a nick surveillance protein. PARP1 is one of the BER complex proteins involved in DNA interruption detection and DNA repair [24]. BER consists of different steps: excision of the base, incision, end processing, and repair synthesis (gap filling and ligation).

Slyskova et al. found that DNA repair capacity (DRC) linked to BER is similar in tumor tissues and adjacent healthy epithelium, suggesting that alterations of BER may be not the crucial events in malignant transformation; however, they could be involved in chemical sensitivity of tumor cells to drugs [25]. 

The MMR system acts against DNA damaging agents in post-replication correction of extra-helical loops and nucleotide mispairs. MMR includes the *MLH1*, *PMS2*, *MSH2*, and *MSH6* genes. Alterations in MMR genes cause microsatellite instability, a mutator phenotype, and a predisposition to colorectal cancer [26]. Moreover, tumors with MMR deficiency show significantly more somatic alterations than MMR efficiency, resulting in an increased neoantigen burden and immunogenicity. Indeed, it has been demonstrated that tumors with MMR deficiency are responsive to anti PD-1 antibodies like pembrolizumab and nivolumab [27]. 

NER consists of about 30 peptides and is involved in the repair of DNA with helix distorting damages, including that caused by UV light, environmental mutagens, and chemotherapeutic agents [28]. The main steps in NER are as follows: recognition of a DNA defect; recruitment of a repair complex; involvement of helicases for DNA repair; incision of the damaged strand, which results in a single-strand fragment of 24–32 nucleotides; DNA synthesis to fill in the gap; and ligation to form the final phosphodiester bond [29]. Indeed, lesions are recognized by XPC-RAD23B, which interacts with TFIIH, a transcription initiation complex, prying the DNA open with an XPD subunit. XPB recruits XPA, RPA, and XPG, allowing the formation of a pre-incision complex. XPA interacts with ERCC1-XPF, making a 5’ incision of the lesion. DNA ligase IIIa/XRCC1 or DNA ligase I concludes the NER process [30].

Slyskova et al. identified alterations of DRC in sporadic colorectal cancer and hypothesized a role of NER in carcinogenesis [31,32].

A double strand break (DSB) is the most lethal damage to the genome that can derive from anti-cancer treatments (e.g., ionizing radiation or the topoisomerase inhibitors) [33] or physiologic pathways (e.g., genetic recombination during meiosis) [34]. 

The HR system is an error-free mechanism that repairs DSBs using a homologous DNA template; during the S/G2 phase of the cell cycle, cyclin-dependent kinases stimulate DNA end resection and activate the HR pathway. The HR process initiates by the end resection generating a long stretch of single-strand DNA from DNA break ends. The HR pathway includes BRCA1 as part of BASC, a large complex linked to genome surveillance composed of MLH1, MSH2, and MSH6 (mismatch repair proteins), an MRN (Mre11–Rad50–Nbs1) complex, and ATM and Bloom (BLM) syndrome helicase [35]. The HR system also involves BRCA2, which forms a complex with Rad51, binding the exposed DNA and permitting Rad51 to load onto the break and assemble the presynaptic filament [36]. The main reactions in HR are catalyzed by the Rad51/RecA family DNA recombinases [37]. 

*RAD51* mutations have been identified in ovarian cancer; specifically, deleterious variants were shown in *RAD51B*, *RAD51C*, and *RAD51D* (nonsense, frameshift, and splice), with a predominance for *RAD51C* and *RAD51D* mutations [38]. Literature data shows that tumors with *RAD51C* and *RAD51D* mutations presented sensitivity to PARP inhibitors, suggesting a novel therapeutic option for this setting of patients [39,40].

The NHEJ system is active during all phases of the cell cycle and ligates DSBs ends without a template. In NHEJ, the DSBs are first recognized by a heterodimer consisting of Ku70 and Ku80 (Ku). The degradation of short regions of the 5’ or 3’ ends by both exonuclease or endonuclease enzymes (e.g., Artemis) is included in the end resection that generates or exposes small regions of microhomology (≤4 nucleotides) between the strands, facilitating end joining. Artemis is recruited with DNA-dependent protein kinase catalytic subunits, which have a high affinity for DNA ends. Nucleotide addition can occur by the Pol X family polymerases. The DNA ligase IV complex, consisting of XRCC4, XLF, and perhaps PAXX, performs the ligation step for either strand of the DSBs. Alternative joining pathways can be involved in DSBs: backup NHEJ (B-NHEJ) makes use of PARP1, PARP2, and ligase III; and microhomology-mediated end-joining (MMEJ) can be considered as a form of B-NHEJ other than alternative end joining (Alt-EJ) [41]. PARP1 could compete with RAD51 and BRCA2 for the further processing of resected ends at DSBs, after the initial phase of end resection. The activation of Alt-EJ is mediated by PARP1, and this determines the aligning of short homologous sequences (i.e., microhomology) in the broken ends of DSBs. The repair pathway mediated by microhomology translates into the generation of small deletions, surrounded by microhomologies. The activity of PARP inhibitors inducing synthetic lethality in BRCA1/2-null cells suggests that PARP1-mediated Alt-EJ compensates for HR in HR-deficient cells [42].

Checkpoints in G1/S, intra-S, and G2/M phases control the progression of cell cycle. The type of DNA lesions activates different DNA damage response proteins [43]. NHEJ is prevalent throughout the cell cycle, whereas HR is dominant during the S and G2 phases, when factors promoting extensive end resection are more effective [44]. Cyclin-dependent kinases favor extensive resection during cell cycle, through enzymes and DNA damage response checkpoint proteins, such as ATM and ataxia telangiectasia and Rad-3 related. Moreover, ATM phosphorylation is demonstrated to be involved in the pathway that allows HR or NHEJ activation [45]. In this context, end processing developing a long 3’ single strand DNA depends on how long DSBs remain unrepaired, which leads to activation of single-strand annealing (SSA). SSA consists of a non-conservative, homology-directed repair pathway that necessitates >20 bp of homology and presents a loss of nucleotides [46]. RAD52 protein is required for the annealing of complementary single strand DNA in the SSA pathway.

Direct DNA repair of base alkylations lesions involves MGMT protein, which repairs the O^6^-methylguanine (highly mutagenic) and human AlkB homologues (*ALKBH1*, *ALKBH2*, and *ALKBH3*). These kinds of lesions can occur during all phases of the cell cycle, so there is no cell cycle regulation for the genes involved in direct repair [47].

Germ line mutations in genes of repair cause a predisposition to cancer. In particular, germ line mutations in *BRCA1* and *BRCA2* are associated mainly with ovarian and breast carcinoma [48], but sporadic cancers also show alterations in *BRCA* genes. Indeed, *BRCA1* interacts with *BRCA2*, and similar phenotypic effects result from *BRCA1* and *BRCA2* mutations [49]. Thus, *BRCA1* and *BRCA2* are two genes that are crucial for repairing DNA damage and for ensuring genomic stability, preventing the accumulation of gross chromosomal rearrangements that would ultimately lead to either cellular apoptosis or tumor formation [50]. 

## 3. Consequence of DNA Repair Deficiencies in Ovarian Cancer 

Based on epidemiologic studies, about 65% to 75% of all cases of hereditary ovarian cancer are caused by gene mutations in *BRCA1* or *BRCA2*. The third major cause of hereditary ovarian cancer is hereditary nonpolyposis colorectal cancer (HNPCC) syndrome, which accounts for an additional 10% to 15% of all inherited cases [51]. HNPCC is caused by mutations in genes involved in the MMR system. 

It is widely acknowledged that HR-deficient ovarian cancers are enriched for high-grade serous histology (Table 1). *BRCA* abnormalities seldom occur in non-high-grade serous ovarian carcinoma subtypes [52]. HR deficiency endows ovarian cancers with a clinical phenotype that is characterized by visceral relapse, a slightly younger age at diagnosis, and a better response to platinum-based chemotherapy, PARP inhibitors, and anthracyclines [42]. 

Usually, only one mutated allele results from inherited germline defects (typically mutations), and loss of the other allele occurs somatically, as in Lynch syndrome (or HNPCC), an autosomal dominant condition that predisposes the patient to cancer development (especially colorectal, ovarian, and endometrial cancer) [53]. Alternatively, sporadic MMR deficient tumors are often due to hypermethylation of the *MLH1* promoter resulting in epigenetic silencing [54].

The distribution of ovarian cancer histotypes in MMR-deficient patients differs considerably from that generally observed: non-serous histologies are more common, and often show endometrioid or a clear cell differentiation (Table 1) [55]. Ovarian endometrioid cancers, accounting for ~10%–25% of all ovarian carcinomas, is predominantly seen in perimenopausal women, and arises from endometriosis, which appears to act as a precursor. Endometrioid histology frequently harbors AT-rich interactive domain 1A (*ARID1A*) mutations, leading to loss of ARID1A protein expression, B-catenin (*CTNNB1*) somatic mutations, *PTEN* mutations, and microsatellite instability [56,57]. In particular, defective mismatch repair protein immunohistochemistry, accounting for the microsatellite instability phenotype, has been reported in 10%–14% of ovarian endometrioid carcinomas. Primarily, loss of *MSH2* and/or *MSH6* accounts for over 50% of MMR-deficient ovarian endometrioid carcinomas [17,58].

Clear-cell ovarian cancer is a rare subtype characterized by a worse prognosis when diagnosed at an advanced stage, due to low chemosensitivity. Howitt and coworkers demonstrated that 10% of clear-cell ovarian cancer exhibited microsatellite instability and roughly 27% *ARID1A* loss [59].

### Immune-Consequence of DNA Repair Defects

The first evidence of a relationship between ovarian cancer and the immune environment was reported by Zhang and coworkers in 2003. The authors demonstrated that the presence of intratumoral T cells correlated with the clinical outcome of advanced ovarian carcinoma [19]. Of note, TIL are more frequently present in serous carcinomas, compared to either endometrioid or clear-cell carcinomas (Table 1). Clarke and coworkers also performed an exploratory analysis in a small case series, observing a significant association between intraepithelial TIL and *BRCA1* mutations or promoter methylation causing loss of expression, mainly in high-grade serous ovarian cancer [60]. Since then, others authors have identified “prominent intraepithelial lymphocytes” as a distinguishing feature of *BRCA1/2*-mutated tumors, with a higher mutational load [61]. 

Although both genes encode proteins that participate in the HR pathway, the reason why germline *BRCA1* mutations seem to confer a higher risk of developing ovarian cancer than germline *BRCA2* mutations is probably related to their earlier and more substantial role in DNA damage response and cell-cycle regulation. Indeed, *BRCA1*-mutant, high-grade serous ovarian cancers present a specific molecular subtype with a distinct gene expression signature, which seems related to specific amplification events at 8q24 and on the X chromosome. Conversely, *BRCA2*-mutant tumors more closely resemble “wild-type” high-grade serous ovarian cancer [62]. Consequently, it seems that *BRCA2*-disrupted tumors, although harboring similar numbers of point mutations, are less immunogenic than *BRCA1*-disrupted tumors [63].

Strickland and coworkers demonstrated that a higher neoantigen load in the *BRCA1/2*-mutated ovarian cancers compared to HR-proficient tumors translates to a significantly higher number of CD3+ TILs compared to HR-proficient tumors. Moreover, HR-proficient tumors showed a lower PD-L1 expression on the surface of intraepithelial and peritumoral immune cells compared to the *BRCA1/2*-mutated tumors, supporting a link between *BRCA1/2*-mutation status, immunogenicity, and improved survival in high grade serous ovarian cancer [64].

With regard to defects in the MMR system, it has been demonstrated that mismatch repair–deficient cancers are associated with 10- to 100-fold more somatic mutations as MMR–proficient cancers, and contain prominent lymphocyte infiltrates, a finding consistent with an immune response. In an unselected series of ovarian clear cell carcinoma, with around 6% of MMR deficiency, peritumoral lymphocytes were more frequent in MMR-deficient tumors [65]. Indeed, it has been seen that an MMR–deficient tumor microenvironment strongly expresses several immune checkpoint ligands, (e.g., PD-1, PD-L1, CTLA-4, LAG-3, and IDO), indicating an immune escape process where their active immune microenvironment is counterbalanced by immune inhibitory signals [66]. 

MMR-deficient tumors were shown to be more frequently resistant to chemotherapy, and in particular to methylating agents and platinum compounds [67]. A possible explanation may be related to the incapability of MMR proteins involved in DNA damage response to recruit ATM/ATR, which in turn leads to cell cycle arrest, DNA repair, or apoptosis [68]. 

Alterations in DNA repair pathways are not the only events that may have immune consequences. In fact, inflammation is the process where reactive oxygen and nitrogen species (RONS) and other mediators, including cytokines, metalloproteinases (MMPs), and PGE2, are produced by inflammatory cells. The same inflammatory signals may, in turn, amplify and perpetuate the inflammatory cascade—e.g., MMPs induce reactive oxygen intermediates, whereas cytokines induce PGE2. The cGAS/STING pathway consists of the activation of a cGAS enzyme by aberrant cytosolic DNA that produces cGAMP, activating the STING protein, leading to the production of pro-inflammatory cytokines, such as type I interferon (IFN), that boost the immune response [69].

Inflammation has the capability to induce the production of HIF-1α in cancer cells, as a consequence of inflammatory cytokines (TNF and IL-1β), prostaglandin (PGE2), and RONS. HIF-1α in turn downregulates MMR proteins, such as MSH2 and MSH6, by displacing c-Myc from *MSH2*/*MSH6* promoters. A potent RONS, hydrogen peroxide, may damage several proteins and enzymes, including MMR members, disrupting their function and ultimately inactivating this DNA-repair pathway. The BER pathway, which serves to repair DNA damage caused by UV exposure and chemotherapeutic agents, appears to be affected by IL-6, which induces hypermethylation in multiple myeloma cells, leading to dysfunction of the key nucleotide excision repair component hHR23B [70]. Moreover, HIF-1α induces microRNA-373, which downregulates the expression of the NER component RAD23B [71].

## 4. Effect of Locoregional Therapeutic Approaches on the Immune System

The aim of locoregional treatments is to deliver higher concentrations of chemotherapy (e.g., cisplatin or paclitaxel) to the peritoneal cavity than that measured in plasma after intravenous administration [72,73], permitting continuous and prolonged exposure of high drug concentrations with lower peak plasma levels over time [74], and thus causing fewer adverse events. Intraperitoneal chemotherapy has been shown to enhance OS and PFS with respect to intravenous administration [75,76], and HIPEC plus complete or optimal interval CRS resulted in longer OS than CRS alone [20].

The proposed advantages of HIPEC over intraperitoneal administration are a single-dose approach with direct observation of intraoperative drug exposure, without the risk of barriers due to postoperative adhesions, and a high-proportion cisplatin dose absorbed by target tumor cells; hyperthermia that has been shown to increase tumor penetration of chemotherapy, and thus enhances cyotoxicity synergistically [77]. This was confirmed by the presence of cisplatin-induced DNA adducts in tumor samples treated with HIPEC [22].

The results obtained with this procedure in terms of improvement in OS can be attributed to the potential interaction between intraperitoneal chemotherapy, the immune system, and inflammatory processes [78,79,80]. In vivo and in vitro studies have shown that the hyperthermic phase of the procedure is characterized by a sharp and predictable increase of inflammatory markers. Cisplatin is a DNA damaging agent, known to cause G2 arrest. It has been seen that HIPEC leads to an increase in Cyclin A1 (up to 53-fold more) and in SSX-4 (up to 30-fold more), with respect to intravenous chemotherapy [79]. Thus, HIPEC causes cell cycle arrest thanks to CDKN1A expression, which in response to DNA damage through p53 activation, binds and inhibits the CDK2/cyclin E complex, preventing the phosphorylation of Rb and thus arresting cancer cells in the G1 phase [81]. Furthermore, serum levels of Interleukine-6 and procalcitonin showed clinically relevant variation during HIPEC [80]. 

Systemic inflammatory indexes, such as the neutrophil-to-lymphocyte ratio (NLR), platelet-to-lymphocyte ratio (PLR), and systemic inflammatory index (SII) (calculated as (platelet count × neutrophil count)/lymphocyte count) have been investigated in several tumors, displaying a potential prognostic and predictive role [82,83,84,85]. In ovarian cancer patients, elevated baseline levels of NLR are associated with a poor prognosis [86,87]. Interestingly, it has been observed that inflammatory markers, such as NLR and SII, may also be predictors of treatment efficacy in ovarian cancer. However, their role has not yet been evaluated in patients undergoing HIPEC [88].

Hypertermia and surgery are stressful procedures for cancer and mesothelial cells. In fact, even if a CA-125 serum marker significantly increases during the peritoneal perfusate, as a consequence of the production of mesothelial cells, it has been demonstrated that there is a trend in its reduction, even if not statistically significant, after this procedure. These data confirm the effectiveness of peritoneal plasma barrier [89,90] in confining the harm produced by the intra-abdominal process to the abdominal cavity [80]. 

Zunino and coworkers investigated the role of hyperthermia mitomycin C and HIPEC (hyperthermia and mitomycin C) in the anticancer immune response. Using murine colon carcinoma cell line CT26, they showed that CT26 cells were killed with all these procedures. However, although mitomycin C and HIPEC treatment caused an activation of dendritic cells (DCs), hyperthermia alone did not. The activated DCs activated T cells in a tumor antigen-dependent manner. The authors also observed a permanent antitumor immune response in mice vaccinated with mitomycine- or HIPEC-treated CT26 cells, through the exposure of Heat Shock Protein 90 (HSP90) on the cell surface of the dying cells [91,92]. These findings showed that the HIPEC procedure not only killed tumor cells, but also induced an efficient anticancer immune response, providing a rationale for using a combination of HIPEC and immunotherapy or intraperitoneal immunotherapy to improve clinical outcome.

## 5. Immune-Modulating Treatment Rationale in Ovarian Cancer 

Immune checkpoint inhibitors, such as anti-PD-1 and anti-PD-L1 antibodies, have proven effective in cancers with a high mutational load, including lung cancer, melanoma, and bladder cancer. A higher mutational load in urothelial cancer was shown to be an independent predictive factor of response to anti-PD-L1 [93,94]. This is a result of bladder tumors producing more tumor-specific neoantigens, which leads to TIL stimulation and PD-1/PD-L1 overexpression. Higher-neoantigen intratumor heterogeneity may result in lower antigen dosage compared to homogeneous tumors with a high clonal neoantigen burden, thus reducing the possibility of identifying T cells reactive to sub-clonal neoantigens and responsive to immune checkpoint inhibitors [95]. In this context, tumors harboring *BRCA* mutations or with a BRCA-like phenotype have a higher probability of producing new neoantigens and a higher mutational load, resulting in more immunogenic tumors. Moreover, it has been demonstrated that PD-1 and PD-L1 are more highly expressed in *BRCA*-mutated tumors than in HR-proficient tumors [64], providing a rationale for treating these tumors with immune checkpoint inhibitors either alone or in combination with PARP inhibitors. Similarly, MMR-deficient tumors are associated with a higher mutational load than MMR-proficient tumors. Indeed, Le and coworkers demonstrated that an immune checkpoint inhibitor, specifically a PD-1 inhibitor, was active against MMR–deficient colorectal cancers [27]. Of note, PD-L1 expression in tumor cells or immune cells is observed in all cases of clear-cell ovarian cancers with microsatellite instability [59], suggesting the usefulness of routine testing of MMR-deficiency in ovarian cancer and opening an alternative therapeutic avenue with immune checkpoint inhibitors for selected ovarian cancer patients. Again, an ARID1A-deficient ovarian cancer cell line, because ARID1A cannot interact anymore with MSH2 during DNA replication, results in increased mutagenesis, increased mutation load, elevated numbers of tumor-infiltrating lymphocytes, and PD-L1 expression. Notably, treatment with anti-PD-L1 antibody seems efficacious in preclinical models bearing ARID1A-deficient but not ARID1A-wild-type ovarian tumors [96].

Promising studies have also been conducted with intraperitoneal immunotherapy. In April 2009, EMA approved the use of intraperitoneal catumaxomab for malignant ascites in patients with epithelial cell adhesion molecule (EpCAM)-positive ovarian and non-gynecological carcinomas after studies confirmed a prolongation in the time of first deterioration of quality of life, median paracentesis-free survival, and overall survival [97,98]. 

Of note, other recent studies focusing on IP immunotherapy with dendritic cell vaccine and cytokine-induced killer cells have demonstrated a significant improvement in quality of life and control of the production of malignant ascites in patients with unresectable peritoneal carcinomatosis [99]. The role of chimeric antigen receptor-engineered T cells (CAR-T cells) in controlling peritoneal carcinomatosis has also been investigated by targeting antigen-specific tumors [100]. A study in mice by Katz and coworkers evaluated tumor response by CAR-T therapy via intraperitoneal or tail-vein injection. The authors reported a 37-fold reduction in peritoneal carcinomatosis in intraperitoneal-treated mice as compared with the tail-vein injection group [101]. Similarly, other studies focusing on peritoneal carcinomatosis from ovarian cancer reported an eradication of tumor growth and a significant improvement in survival in the intraperitoneal CAR-T cell-treated mice models compared with control groups [102,103,104]. 

## 6. Conclusions

Substantial progress has been achieved in understanding the biology of ovarian cancer in the last few years. Ovarian cancer is strongly linked to defects in DNA repair mechanisms. It was recently demonstrated that different ovarian cancer histotypes may have different immunogenicity, following the recognition of six molecular subgroups, including an “immunoreactive” subtype, by the Cancer Genome Atlas Research Network (TCGA) [13]. In this context, the combination of immunotherapy and PARP inhibition in HR- or MMR-deficient ovarian cancers is a potentially interesting hypothesis. Moreover, there is evidence to show that HIPEC has an effect on immune response by activating the mediators of the non-specific innate immune system to kill tumor cells. However, HIPEC also induces the adaptive immune system to create an efficient anticancer immune response, protecting patients on a long-term basis. Within this context, it would be interesting to carry out studies on intraperitoneal immunotherapy, including combinations with PARP inhibitors, in patients harboring defects in DNA repair pathways.

## Figures and Tables

**Table 1:** Characteristics of different ovarian cancer histological types.

Clinical Characteristics	High-grade Serous	Low-grade Serous	Clear Cell	Endometrioid	Mucinous
**Prevalence**	65%–70%	3%	5%–10%	10%–15%	2%–8%
**Hereditary risk**	18%–20% present germline BRCA1/2 mutations	unknown	unknown	10%–14% endometrioid tumors are associated with HNPCC syndrome	unknown
**Stage at diagnosis**	Advanced	Early Advanced	Early	Early	Early
**Genetic alterations**	p53 p16 pRb pathway Homologous recombination defects (BRCA1/2, RAD51)	BRAF or KRAS	HNF-1β IL6/JAK2/STAT3 PI3K MSI ARID1A	PTEN β-Catenin KRAS MSI ARID1A	K-ras c-MYC HER2
**Chemotherapy response**	80%	26–28%	15%	unknown	15%
**Immune infiltrate**	High, more commonly associated with BRCA1 defects	Low	Generally low, higher when associated with MSI	Generally low, higher when associated with MSI	Low

## References

[1] GLOBOCAN. http://gco.iarc.fr/.

[2] Vergote I., Trope C.G., Amant F., Kristensen G.B., Ehlen T., Johnson N., Verheijen R.H., van der Burg M.E., Lacave A.J., Panici P.B. (2010). Neoadjuvant chemotherapy or primary surgery in stage IIIC or IV ovarian cancer. N. Engl. J. Med..

[3] Armstrong D.K., Bundy B., Wenzel L., Huang H.Q., Baergen R., Lele S., Copeland L.J., Walker J.L., Burger R.A. (2006). Intraperitoneal cisplatin and paclitaxel in ovarian cancer. N. Engl. J. Med..

[4] Prat J., D’Angelo E., Espinosa I. (2018). Ovarian carcinomas: At least five different diseases with distinct histological features and molecular genetics. Hum. Pathol..

[5] McCluggage W.G. (2011). Morphological subtypes of ovarian carcinoma: A review with emphasis on new developments and pathogenesis. Pathology.

[6] Kurman R.J., Carcangiu M.L., Herrington C.S., Young R.H. (2014). WHO classification of tumours of female reproductive organs, 4th ed.

[7] Karabuk E., Kose M.F., Hizli D., Taşkin S., Karadağ B., Turan T., Boran N., Ozfuttu A., Ortaç U.F. (2013). Comparison of advanced stage mucinous epithelial ovarian cancer and serous epithelial ovarian cancer with regard to chemosensitivity and survival outcome: A matched case-control study. J. Gynecol. Oncol..

[8] Risch H.A., McLaughlin J.R., Cole D.E., Rosen B., Bradley L., Fan I., Tang J., Li S., Zhang S., Shaw P.A., Narod S.A. (2006). Population BRCA1 and BRCA2 mutation frequencies and cancer penetrances: A kin-cohort study in Ontario, Canada. J. Natl. Cancer Inst..

[9] Hanahan D., Weinberg R.A. (2011). Hallmarks of cancer: The next generation. Cell.

[10] Dietlein F., Thelen L., Reinhardt H.C. (2014). Cancer-specific defects in DNA repair pathways as targets for personalized therapeutic approaches. Trends Genet..

[11] Press J.Z., De Luca A., Boyd N., Young S., Troussard A., Ridge Y., Kaurah P., Kalloger S.E., Blood K.A., Smith M. (2008). Ovarian carcinomas with genetic and epigenetic BRCA1 loss have distinct molecular abnormalities. BMC Cancer.

[12] Hennessy B.T., Timms K.M., Carey M.S., Gutin A., Meyer L.A., Flake D.D., Abkevich V., Potter J., Pruss D., Glenn P. (2010). Somatic mutations in BRCA1 and BRCA2 could expand the number of patients that benefit from poly (ADP ribose) polymerase inhibitors in ovarian cancer. J. Clin. Oncol..

[13] Cancer Genome Atlas Research Network (2011). Integrated Genomic Analyses Of Ovarian Carcinoma. Nature.

[14] Hilton J.L., Geisler J.P., Rathe J.A., Rathe J.A., Hattermann-Zogg M.A., DeYoung B., Buller R.E. (2002). Inactivation of BRCA1 and BRCA2 in ovarian cancer. J. Natl. Cancer Inst..

[15] Geisler J.P., Hatterman-Zogg M.A., Rathe J.A., Buller R.E. (2002). Frequency of BRCA1 dysfunction in ovarian cancer. J. Natl. Cancer Inst..

[16] Mukhopadhyay A., Curtin N., Plummer R., Edmondson R.J. (2011). PARP inhibitors and epithelial ovarian cancer: An approach to targeted chemotherapy and personalised medicine. Br. J. Obstet. Gynaecol..

[17] Aysal A., Karnezis A., Medhi I., Grenert J.P., Zaloudek C.J., Rabban J.T. (2012). Ovarian endometrioid adenocarcinoma: Incidence and clinical significance of the morphologic and immunohistochemical markers of mismatch repair protein defects and tumor microsatellite instability. Am. J. Surg. Pathol..

[18] Morice P., Gouy S., Leary A. (2019). Mucinous Ovarian Carcinoma. N. Engl. J. Med..

[19] Zhang L., Conejo-Garcia J.R., Katsaros D., Katsaros D., Gimotty P.A., Massobrio M., Regnani G., Makrigiannakis A., Gray H., Schlienger K. (2003). Intratumoral T cells, recurrence, and survival in epithelial ovarian cancer. N. Engl. J. Med..

[20] Van Driel W.J., Koole S.N., Sikorska K., Schagen van Leeuwen J.H., Schreuder H.W.R., Hermans R.H.M., de Hingh I.H.J.T., van der Velden J., Arts H.J., Massuger L.F.A.G. (2018). Hyperthermic Intraperitoneal Chemotherapy in Ovarian Cancer. N. Engl. J. Med..

[21] Spiliotis J., Halkia E., Lianos E., Kalantzi N., Grivas A., Efstathiou E., Giassas S. (2015). Cytoreductive surgery and HIPEC in recurrent epithelial ovariancancer: A prospective randomized phase III study. Ann. Surg. Oncol..

[22] Zivanovic O., Abramian A., Kullmann M., Fuhrmann C., Coch C., Hoeller T., Ruehs H., Keyver-Paik M.D., Rudlowski C., Weber S. (2015). HIPEC ROC I: A phase I study of cisplatin administered as hyperthermic intraoperative intraperitoneal chemoperfusion followed by postoperative intravenous platinum-based chemotherapy in patients with platinum-sensitive recurrent epithelial ovarian cancer. Int. J. Cancer.

[23] Evans A.R., Limp-Foster M., Kelley M.R. (2000). Going APE over ref-1. Mutat. Res..

[24] Carter R.J., Parsons J.L. (2016). Base Excision Repair, a Pathway Regulated by Posttranslational Modifications. Mol. Cell. Biol..

[25] Slyskova J., Korenkova V., Collins A.R., Prochazka P., Vodickova L., Svec J., Lipska L., Levy M., Schneiderova M., Liska V. (2012). Functional, genetic, and epigenetic aspects of base and nucleotide excision repair in colorectal carcinomas. Clin. Cancer Res..

[26] Bernstein C., Bernstein H., Payne C.M., Garewal H. (2002). DNA repair/pro-apoptotic dual-role proteins in five major DNA repair pathways: Fail-safe protection against carcinogenesis. Mutat. Res..

[27] Le D.T., Uram J.N., Wang H., Bartlett B.R., Kemberling H., Eyring A.D., Skora A.D., Luber B.S., Azad N.S., Laheru D. (2015). PD-1 Blockade in Tumors with Mismatch-Repair Deficiency. N. Engl. J. Med..

[28] Geske F.J., Nelson A.C., Lieberman R., Strange R., Sun T., Gerschenson L.E. (2000). DNA repair is activated in early stages of p53-induced apoptosis. Cell Death Differ..

[29] Spivak G. (2015). Nucleotide excision repair in humans. DNA Repair.

[30] Schärer O.D. (2013). Nucleotide excision repair in eukaryotes. Cold Spring Harb. Perspect. Biol..

[31] Slyskova J., Naccarati A., Pardini B., Polakova V., Vodickova L., Smerhovsky Z., Levy M., Lipska L., Liska V., Vodicka P. (2012). Differences in nucleotide excision repair capacity between newly diagnosed colorectal cancer patients and healthy controls. Mutagenesis.

[32] Slyskova J., Cordero F., Pardini B., Korenkova V., Vymetalkova V., Bielik L., Vodickova L., Pitule P., Liska V., Matejka (2015). Post-treatment recovery of suboptimal DNA repair capacity and gene expression levels in colorectal cancer patients. Mol. Carcinog..

[33] Helleday T., Petermann E., Lundin C., Hodgson B., Sharma R.A. (2008). DNA repair pathways as targets for cancer therapy. Nat. Rev. Cancer..

[34] Neale M.J., Keeney S. (2006). Clarifying the mechanics of DNA strand exchange in meiotic recombination. Nature.

[35] Gudmundsdottir K., Ashworth A. (2006). The roles of BRCA1 and BRCA2 and associated proteins in the maintenance of genomic stability. Oncogene.

[36] Yang H., Jeffrey P.D., Miller J., Kinnucan E., Sun Y., Thoma N.H., Zheng N., Chen P.L., Lee W.H., Pavletich N.P. (2002). BRCA2 function in DNA binding and recombination from a BRCA2-DSS1-ssDNA structure. Science.

[37] Kowalczykowski S.C. (2015). An overview of the molecular mechanisms of recombinational DNA repair. Cold Spring Harb. Perspect. Biol..

[38] Golmard L., Castéra L., Krieger S., Moncoutier V., Abidallah K., Tenreiro H., Laugé A., Tarabeux J., Millot G.A., Nicolas A. (2017). Contribution of germline deleterious variants in the RAD51 paralogs to breast and ovarian cancers. Eur. J. Hum. Genet..

[39] Loveday C., Turnbull C., Ramsay E., Hughes D., Ruark E., Frankum J.R., Bowden G., Kalmyrzaev B., Warren-Perry M., Snape K. (2011). Germline mutations in RAD51D confer susceptibility to ovarian cancer. Nat. Genet..

[40] Min A., Im S.A., Yoon Y.K., Song S.H., Nam H.J., Hur H.S., Kim H.P., Lee K.H., Han S.W., Oh D.Y. (2013). RAD51C-deficient cancer cells are highly sensitive to the PARP inhibitor olaparib. Mol. Cancer Ther..

[41] Durante M., Bedford J.S., Chen D.J., Conrad S., Cornforth M.N., Natarajan A.T., van Gent D.C., Obe G. (2013). From DNA damage to chromosome aberrations: Joining the break. Mutat. Res..

[42] Vanderstichele A., Busschaert P., Olbrecht S., Lambrechts D., Vergote I. (2017). Genomic signatures as predictive biomarkers of homologous recombination deficiency in ovarian cancer. Eur. J. Cancer.

[43] Cimprich K.A., Cortez D. (2008). ATR: An essential regulator of genome integrity. Nat. Rev. Mol. Cell Biol..

[44] Chang H.H.Y., Pannunzio N.R., Adachi N., Lieber M.R. (2017). Non-homologous DNA end joining and alternative pathways to double-strand break repair. Nat. Rev. Mol. Cell Biol..

[45] Zhou Y., Lee J.H., Jiang W., Crowe J.L., Zha S., Paull T.T. (2017). Regulation of the DNA damage response by DNA-PKcs inhibitory phosphorylation of ATM. Mol. Cell.

[46] Bhargava R., Onyango D.O., Stark J.M. (2016). Regulation of single-strand annealing and its role in genome maintenance. Trends Genet..

[47] Mjelle R., Hegre S.A., Aas P.A., Slupphaug G., Drabløs F., Saetrom P., Krokan H.E. (2015). Cell cycle regulation of human DNA repair and chromatin remodeling genes. DNA Repair.

[48] Goyal G., Fan T., Silberstein P.T. (2016). Hereditary cancer syndromes: Utilizing DNA repair deficiency as therapeutic target. Fam. Cancer.

[49] Welcsh P.L., Owens K.N., King M.C. (2000). Insights into the functions of BRCA1 and BRCA2. Trends Genet..

[50] Yu V.P., Koehler M., Steinlein C., Schmid M., Hanakahi L.A., van Gool A.J., West S.C., Venkitaraman A.R. (2000). Gross chromosomal rearrangements and genetic exchange between nonhomologous chromosomes following BRCA2 inactivation. Genes Dev..

[51] Bewtra C., Watson P., Conway T., Conway T., Read-Hippee C., Lynch H.T. (1992). Hereditary ovarian cancer: A clinicopathological study. Int. J. Gynecol. Pathol..

[52] McAlpine J.N., Porter H., Köbel M., Nelson B.H., Prentice L.M., Kalloger S.E., Senz J., Milne K., Ding J., Shah S.P., Huntsman D.G., Gilks C.B. (2012). BRCA1 and BRCA2 mutations correlate with TP53 abnormalities and presence of immune cell infiltrates in ovarian high-grade serous carcinoma. Mod. Pathol..

[53] Peltomäki P. (2005). Peltomäki. Lynch syndrome genes. Familial Cancer.

[54] Beggs A.D., Domingo E., Abulafi M., Hodgson S.V., Tomlinson I.P.M. (2013). A study of genomic instability in early preneoplastic colonic lesions. Oncogene.

[55] Ketabi Z., Bartuma K., Bernstein I., Malander S., Grönberg H., Björck E., Holck S., Nilbert M. (2011). Ovarian cancer linked to Lynch syndrome typically presents as early-onset, non-serous epithelial tumors. Gynecol. Oncol..

[56] Wiegand K.C., Shah S.P., Al-Agha O.M., Zhao Y., Tse K., Zeng T., Senz J., McConechy M.K., Anglesio M.S., Kalloger S.E. (2010). ARID1A mutations in endometriosis-associated ovarian carcinomas. N. Engl. J. Med..

[57] Parra-Herran C., Lerner-Ellis J., Xu B., Khalouei S., Bassiouny D., Cesari M., Ismiil N., Nofech-Mozes S. (2017). Molecular-based classification algorithm for endometrial carcinoma categorizes ovarian endometrioid carcinoma into prognostically significant groups. Mod. Pathol..

[58] Rambau P.F., Duggan M.A., Ghatage P., Warfa K., Steed H., Perrier R., Kelemen L.E., Köbel M. (2016). Significant frequency of MSH2/MSH6 abnormality in ovarian endometrioid carcinoma supports histotype-specific Lynch syndrome screening in ovarian carcinomas. Histopathology.

[59] Howitt B.E., Strickland K.C., Sholl L.M., Rodig S., Ritterhouse L.L., Chowdhury D., D’Andrea A.D., Matulonis U.A., Konstantinopoulos P.A. (2017). Clear cell ovarian cancers with microsatellite instability: A unique subset of ovarian cancers with increased tumor-infiltrating lymphocytes and PD-1/PDL1 expression. Oncoimmunology.

[60] Clarke B., Tinker A.V., Lee C.H., Subramanian S., van de Rijn M., Turbin D., Kalloger S., Han G., Ceballos K., Cadungog M.G. (2009). Intraepithelial T cells and prognosis in ovarian carcinoma: Novel associations with stage, tumor type, and BRCA1 loss. Mod. Pathol..

[61] Birkbak N.J., Kochupurakkal B., Izarzugaza J.M., Eklund A.C., Li Y., Liu J., Szallasi Z., Matulonis U.A., Richardson A.L., Iglehart J.D. (2013). Tumor mutation burden forecasts outcome in ovarian cancer with BRCA1 or BRCA2 mutations. PLoS ONE.

[62] George J., Alsop K., Etemadmoghadam D., Hondow H., Mikeska T., Dobrovic A., deFazio A., Smyth G.K., Levine D.A., Mitchell G. (2013). Non equivalent gene expression and copy number alterations in high-grade serous ovarian cancers with BRCA1 and BRCA2 mutations. Clin. Cancer Res..

[63] Nelson B.H. (2015). New insights into tumor immunity revealed by the unique genetic and genomic aspects of ovarian cancer. Curr. Opin. Immunol..

[64] Strickland K.C., Howitt B.E., Shukla S.A., Rodig S., Ritterhouse L.L., Liu J.F., Garber J.E., Chowdhury D., Wu C.J., D’Andrea A.D., Matulonis U.A., Konstantinopoulos P.A. (2016). Association and prognostic significance of BRCA1/2-mutation status with neoantigen load, number of tumor-infiltrating lymphocytes and expression of PD-1/PD-L1 in high grade serous ovarian cancer. Oncotarget.

[65] Bennett J.A., Morales-Oyarvide V., Campbell S., Longacre T.A., Oliva E. (2016). Mismatch Repair Protein Expression in Clear Cell Carcinoma of the Ovary: Incidence and Morphologic Associations in 109 Cases. Am. J. Surg. Pathol..

[66] Llosa N.J., Cruise M., Tam A., Wicks E.C., Hechenbleikner E.M., Taube J.M., Blosser R.L., Fan H., Wang H., Luber B.S. (2015). The vigorous immune microenvironment of microsatellite instable colon cancer is balanced by multiple counter-inhibitory checkpoints. Cancer Discov..

[67] Hewish M., Lord C.J., Martin S.A., Cunningham D., Ashworth A. (2010). Mismatch repair deficient colorectal cancer in the era of personalized treatment. Nat. Rev. Clin. Oncol..

[68] Viale G., Trapani D., Curigliano G. (2017). Mismatch Repair Deficiency as a Predictive Biomarker for Immunotherapy Efficacy. Biomed. Res. Int..

[69] Bose D. (2017). cGAS/STING Pathway in Cancer: Jekyll and Hyde Story of Cancer Immune Response. Int. J. Mol. Sci..

[70] Peng B., Hodge D.R., Thomas S.B., Cherry J.M., Munroe D.J., Pompeia C., Xiao W., Farrar W.L. (2005). Epigenetic silencing of the human nucleotide excision repair gene, hHR23B, in interleukin-6-responsive multiple myeloma KAS-6/1 cells. J. Biol. Chem..

[71] Colotta F., Allavena P., Sica A., Garlanda C., Mantovani A. (2009). Cancer-related inflammation, the seventh hallmark of cancer: Links to genetic instability. Carcinogenesis.

[72] Lopez J.A., Krikorian J.G., Reich S.D., Smyth R.D., Lee F.H., Issell B.F. (1985). Clinical pharmacology of intraperitoneal cisplatin. Gynecol. Oncol..

[73] Francis P., Rowinsky E., Schneider J., Hakes T., Hoskins W., Markman M. (1995). Phase I feasibility and pharmacologic study of weekly intraperitoneal paclitaxel: A Gynecologic Oncology Group pilot study. J. Clin. Oncol..

[74] Tummala M.K., Alagarsamy S., McGuire W.P. (1987). Intraperitoneal chemotherapy: Standard of care for patients with minimal residual stage III ovarian cancer?. Expert Rev. Anticancer Ther..

[75] Tewari D., Java J.J., Salani R., Armstrong D.K., Markman M., Herzog T., Monk B.J., Chan J.K. (2015). Long-term survival advantage and prognostic factors associated with intraperitoneal chemotherapy treatment in advanced ovarian cancer: A gynecologic oncology group study. J. Clin. Oncol..

[76] Jaaback K., Johnson N., Lawrie T.A. (2016). Intraperitoneal chemotherapy for the initial management of primary epithelial ovarian cancer. Cochrane Database Syst. Rev..

[77] Panteix G., Beaujard A., Garbit F., Chaduiron-Faye C., Guillaumont M., Gilly F., Baltassat P., Bressolle F. (2002). Population pharmacokinetics of cisplatin in patients with advanced ovarian cancer during intraperitoneal hyperthermia chemotherapy. Anticancer Res..

[78] Yu H., Dong R., Lu Y., Yang X., Chen C., Zhang Z., Peng M. (2017). Short-term postoperative cognitive disfunction and inflammatory response in patients undergoing cytoreductive surgery and hyperthermic intraperitoneal chemotherapy: A pilot study. Mediat. Inflamm..

[79] Roth L., Breuer E., Gupta A., Graf R., Clavien P., Lehmann K. The impact of HIPEC on anticancer immune response. Proceedings of the ESMO Immuno-Oncology Congress 2018.

[80] Coccolini F., Corbella D., Finazzi P., Brambillasca P., Benigni A., Prussiani V., Ceresoli M., Manfredi R., Poiasina E., Bertoli P. (2016). Time course of cytokines, hemodynamic and metabolic parameters during hyperthermic intraperitoneal chemotherapy. Minerva Anestesiol..

[81] Muenyi C.S., Trivedi A.P., Helm C.W., States J.C. (2014). Cisplatin plus sodium arsenite and hyperthermia induces pseudo-G1 associated apoptotic cell death in ovarian cancer cells. Toxicol. Sci..

[82] Lolli C., Caffo O., Scarpi E., Aieta M., Conteduca V., Maines F., Bianchi E., Massari F., Veccia A., Chiuri V.E., Facchini G., de Giorgi U. (2016). Systemic Immune-Inflammation Index Predicts the Clinical Outcome in Patients with mCRPC Treated with Abiraterone. Front. Pharmacol..

[83] Rossi L., Santoni M., Crabb S.J., Scarpi E., Burattini L., Chau C., Bianchi E., Savini A., Burgio S.L., Conti A. (2015). High neutrophil-to-lymphocyte ratio persistent during first-line chemotherapy predicts poor clinical outcome in patients with advanced urothelial cancer. Ann. Surg. Oncol..

[84] Lolli C., Basso U., Derosa L., Scarpi E., Sava T., Santoni M., Crabb S.J., Massari F., Aieta M., Conteduca V. (2016). Systemic immune-inflammation index predicts the clinical outcome in patients with metastatic renal cell cancer treated with sunitinib. Oncotarget.

[85] Passardi A., Scarpi E., Cavanna L., Dall’Agata M., Tassinari D., Leo S., Bernardini I., Gelsomino F., Tamberi S., Brandes A.A., Tenti E. (2016). Inflammatory indexes as predictors of prognosis and bevacizumab efficacy in patients with metastatic colorectal cancer. Oncotarget.

[86] Yang Z., Gu J.H., Guo C.S., Li X.H., Yang W.C. (2017). Preoperative neutrophil-to-lymphocyte ratio is a predictor of survival of epithelial ovarian cancer: A systematic review and meta-analysis of observational studies. Oncotarget.

[87] Huang Q.T., Zhou L., Zeng W.J., Ma Q.Q., Wang W., Zhong M., Yu Y.H. (2017). Prognostic Significance of Neutrophil-to-Lymphocyte Ratio in Ovarian Cancer: A Systematic Review and Meta-Analysis of Observational Studies. Cell. Physiol. Biochem..

[88] Farolfi A., Petrone M., Scarpi E., Gallà V., Greco F., Casanova C., Longo L., Cormio G., Orditura M., Bologna A. (2018). Inflammatory indexes as predictive of prognosis and bevacizumab efficacy in ovarian cancer. A multicentre retrospective analysis from the MITO group (MITO 24). Target Oncol..

[89] Glehen O., Stuart O.A., Mohamed F., Sugarbaker P.H. (2004). Hyperthermia modifies pharmacokinetics and tissue distribution of intraperitoneal melphalan in a rat model. Cancer Chemother. Pharmacol..

[90] Chabner B.A., Collins J.M. (1990). Cancer Chemotherapy: Principles and Practice.

[91] Zunino B., Ricci J.E. (2016). Hyperthermic intra-peritoneal chemotherapy and anticancer immune response. Oncoimmunology.

[92] Zunino B., Rubio-Patiño C., Villa E., Meynet O., Proics E., Cornille A., Pommier S., Mondragón L., Chiche J., Bereder J.M. (2016). Hyperthermic intraperitoneal chemotherapy leads to an anticancer immune response via exposure of cell surface heat shock protein 90. Oncogene.

[93] Rosenberg J.E., Hoffman-Censits J., Powles T., van der Heijden M.S., Balar A.V., Necchi A., Dawson N., O’Donnell P.H., Balmanoukian A., Loriot Y. (2016). Atezolizumab in patients with locally advanced and metastatic urothelial carcinoma who have progressed following treatment with platinum-based chemotherapy: A single-arm, multicentre, phase 2 trial. Lancet.

[94] Balar A.V., Galsky M.D., Rosenberg J.E., Powles T., Petrylak D.P., Bellmunt J., Loriot Y., Necchi A., Hoffman-Censits J., Perez-Gracia J.L. (2016). Atezolizumab as first-line treatment in cisplatin-ineligible patients with locally advanced and metastatic urothelial carcinoma: A single-arm, multicentre, phase 2 trial. Lancet.

[95] McGranahan N., Furness A.J., Rosenthal R., Ramskov S., Lyngaa R., Saini S.K., Jamal-Hanjani M., Wilson G.A., Birkbak N.J., Hiley C.T. (2016). Clonal neoantigens elicit T cell immunoreactivity and sensitivity to immune checkpoint blockade. Science.

[96] Shen J., Ju Z., Zhao W., Wang L., Peng Y., Ge Z., Nagel Z., Zou J., Wang C., Kapoor P. (2018). ARID1A deficiency promotes mutability and potentiates therapeutic antitumor immunity unleashed by immune checkpoint blockade. Nat. Med..

[97] Wimberger P., Gilet H., Gonschior A.K., Heiss M.M., Moehler M., Oskay-Oezcelik G., Al-Batran S.E., Schmalfeldt B., Schmittel A., Schulze E. (2012). Deterioration in quality of life (QoL) in patients with malignant ascites: Results from a phase II/III study comparing paracentesis plus catumaxomab with paracentesis alone. Ann. Oncol..

[98] Heiss M.M., Murawa P., Koralewski P., Kutarska E., Kolesnik O.O., Ivanchenko V.V., Dudnichenko A.S., Aleknaviciene B., Razbadauskas A., Gore M. (2010). The trifunctional antibody catumaxomab for the treatment of malignant ascites due to epithelial cancer: Results of a prospective randomized phase II/III trial. Int. J. Cancer.

[99] Ai Y.Q., Cai K., Hu J.H., Jiang L.W., Gao Y.R., Zhao H., Jia S.C. (2014). The clinical effects of dendritic cell vaccines combined with cytokine-induced killer cells intraperitoneal injected on patients with malignant ascites. Int. J. Clin. Exp. Med..

[100] Morano W.F., Aggarwal A., Love P., Richard S.D., Esquivel J., Bowne W.B. (2016). Intraperitoneal immunotherapy: Historical perspectives and modern therapy. Cancer Gene Ther..

[101] Katz S.C., Point G.R., Cunetta M., Thorn M., Guha P., Espat N.J., Boutros C., Hanna N., Junghans R.P. (2016). Regional CAR-T cell infusions for peritoneal carcinomatosis are superior to systemic delivery. Cancer Gene Ther..

[102] Koneru M., Purdon T.J., Spriggs D., Koneru S., Brentjens R.J. (2015). IL-12 secreting tumor-targeted chimeric antigen receptor T cells eradicate ovarian tumors in vivo. Oncoimmunology.

[103] Hong H., Brown C.E., Ostberg J.R., Priceman S.J., Chang W.C., Weng L., Lin P., Wakabayashi M.T., Jensen M.C., Forman S.J. (2016). Cell Adhesion Molecule-Specific Chimeric Antigen Receptor-Redirected Human T Cells Exhibit Specific and Efficient Antitumor Activity against Human Ovarian Cancer in Mice. PLoS ONE.

[104] Dobrzanski M.J., Rewers-Felkins K.A., Quinlin I.S., Samad K.A., Phillips C.A., Robinson W., Dobrzanski D.J., Wright S.E. (2009). Autologous MUC1-specific Th1 effector cell immunotherapy induces differential levels of systemic TReg cell subpopulations that result in increased ovarian cancer patient survival. Clin. Immunol..

